# Data sharing and data governance in sub-Saharan Africa: Perspectives from researchers and scientists engaged in data-intensive research

**DOI:** 10.17159/sajs.2023/15129

**Published:** 2023-05-30

**Authors:** Siti M. Kabanda, Nezerith Cengiz, Kanshukan Rajaratnam, Bruce W. Watson, Qunita Brown, Tonya M. Esterhuizen, Keymanthri Moodley

**Affiliations:** 1 Centre for Medical Ethics and Law, Faculty of Medicine and Health Sciences, Stellenbosch University, Cape Town, South Africa; 2 School for Data Science and Computational Thinking, Stellenbosch University, Stellenbosch, South Africa; 3 Division of Epidemiology and Biostatistics, Faculty of Medicine and Health Sciences, Stellenbosch University, Cape Town, South Africa

**Keywords:** big data, data governance, sub-Saharan Africa, researchers, scientists, data transfer agreements, data sharing

## Abstract

The data ecosystem is complex and involves multiple stakeholders. Researchers and scientists engaging in data-intensive research collect, analyse, store, manage and share large volumes of data. Consequently, capturing researchers’ and scientists’ views from multidisciplinary fields on data use, sharing and governance adds an important African perspective to emerging debates. We conducted a descriptive cross-sectional survey and received 160 responses from researchers and scientists representing 43 sub-Saharan African countries. Whilst most respondents were satisfied with institutional data storage processes, 40% indicated that their organisations or institutions did not have a formally established process for storing data beyond the life cycle of the project. Willingness to share data was generally high, but increased when data privacy was ensured. Robust governance frameworks increased the willingness to share, as did the regulation of access to data on shared platforms. Incentivising data sharing remains controversial. Respondents were satisfied with exchanging their data for co-authorship on publications (89.4%) and collaboration on projects (77.6%). However, respondents were split almost equally in terms of sharing their data for commercial gain. Regarding the process of managing data, 40.6% indicated that their organisations do not provide training on best practices for data management. This could be related to a lack of resources, chronic institutional under-investment, and suboptimal research training and mentorship in sub-Saharan Africa. The sustainability of data sharing may require ethical incentive structures to further encourage researchers and scientists. Tangible infrastructure to facilitate such sharing is a prerequisite. Capacity development in data governance for researchers and scientists is sorely needed.

**Significance:**

Data sharing is necessary to advance science, yet there are many constraints. In this study, we explored factors that promote a willingness to share, as well as constraining factors. Seeking potential solutions to improve data sharing is a scientific and ethical imperative. The standardisation of basic data sharing and data transfer agreements, and the development of a Data Access Committee will strengthen data governance and facilitate responsible data sharing in sub-Saharan Africa. Funders, institutions, researchers and scientists ought to jointly contribute to fair and equitable data use and sharing during and beyond the life cycle of research projects.

## Background

High-quality and accurate data generated via research have enormous transformative potential for evidence-based decision-making, together with data analytics that helps to improve the tracking of targets that have been put in place.^1,2^ Such advantages, which emanate from the digital revolution, are embodied as velocity, veracity and variability.^3–5^ The consideration of transparency, sharing, governance and management frameworks regarding big data become more challenging in the context of volume, velocity and variety. High-quality data create the foundation of science, regardless of volume (small data or big data), whilst also serving a vital role in informing sound decision-making for optimal action.^6^ As data become a focal point of innovative scientific discovery, data sharing by researchers and scientists has become a critical aspect of scientific advancement.^7^ Data sharing is described as the act of providing access by transferring data in a form that can be used by other individuals.^6,8^ Its prominence in current research debates is premised on open science, which is intended to make data and scientific research widely accessible.^7,9^ This is especially important given that most published articles are not available to people without a personal or institutional subscription, and most data are not made available on public repositories.^10^ As a result, the open science movement has the potential to revolutionise scientific research and improve its transparency and potential for collaboration.^10,11^ Additionally, this encourages researchers and scientists to share their data with others, which can lead to numerous benefits, such as increased scientific reproducibility, robustness and new opportunities for collaboration, thereby enriching the potential to inform interventions or policy decisions.^7,9^ Various initiatives, such as the Transparency and Openness Promotion (TOP) guidelines and the findable, accessible, interoperable and reusable (FAIR) principles, offer guidance for the improved clarity and reproducibility of research.^10,12^ By funding replication studies and recognising and crediting their efforts, researchers can be incentivised to engage in open science practices that can promote transparency, collaboration and innovation in scientific research.^10,13^ Various stakeholders, publishers, funders, custodians of data repositories, tertiary and research institutions, and librarians play a pivotal role in developing structures and systems that support and promote data sharing.^14,15^

Data-sharing policies, such as the Bermuda Principles, the Fort Lauderdale Principles and the Global Alliance for Genomics and Health, expose key principles on open access to genome sequence data^16–18^ with the aim of accelerating advances in science by supporting the free and unrestricted use of such data^18^. The adoption of access policies for publicly funded research has replaced the previous divisive lack of consensus amongst funding agencies and research institutions.^19^

Despite the benefits of data sharing in open science, many researchers and scientists remain reluctant to share their data. This stance is driven by privacy or intellectual property concerns, the historical misuse of data, and concerns of being scooped.^20–22^ Kim et al.^23^ conducted a survey-based descriptive study on the data-sharing attitudes and practices of researchers in Korean government research institutes. From their work, the most common reasons for withholding data included time- and effort-intensive requirements to organise data, followed by concerns about data ownership and lack of reward or recognition for data sharing.^23^ Additionally, Kim et al.^23^ found that respondents had concerns about sharing data that contained sensitive information or where there were potential errors within the data. The degree to which scientists or researchers share or withhold data is not solely a personal choice, as institutional and national factors greatly impact data sharing. For instance, in the context of laws, regulations and policies, restrictions may apply to data sources that are copyrighted and may prohibit the publication of certain types of data (i.e. medical records).^7,24^

Furthermore, data transfer agreements (DTAs) govern the transfer of identifiable human participant data, where voluntary and informed consent have been obtained from participants.^25,26^ Both material transfer agreements (MTAs) and DTAs contractually govern biological material and data transfer between parties to safeguard the interests of stakeholders.^25,26^ These contractual agreements outline the specific purpose(s) for which the data may be used, as well as the related protections, rights and obligations of stakeholders and collaborators. Despite the important role that MTAs and DTAs play in bio-sample and data governance, these agreements are occasionally perceived as an impediment to data sharing, given their complexity and associated bureaucracy.^27^ As a result, it is important to develop strategies and policies to promote effective data sharing, whilst simultaneously maintaining privacy and confidentiality. Although data-sharing practices vary across fields, data-sharing perceptions and experiences can be similar.^28^ In a study conducted by Pujol Priego et al.^28^, researchers in physics, astronomy, life sciences and computer science recognised the benefits of having access to others’ data. However, when compared to physics and astronomy researchers, many researchers in life sciences were less eager to share their data. The reluctance to share data in life sciences could depend on ethical and cultural limitations, especially amongst scientists who work with human participants.^7,29^ The difference in perceptions and practices of data sharing across scientific fields is highly determinative in the fields of life sciences, astronomy and physics due to their long-standing tradition of engagement with large volumes of data compared to other fields.^28^ Nonetheless, most researchers and scientists worldwide have a positive attitude towards data sharing^7^, yet those in low- and middle-income countries (LMICs) face more challenges in this regard.

Various studies illustrate these challenges in LMICs, particularly in sub-Saharan Africa (SSA).^30^ A study by Bangani and Moyo^31^ found that limited resources increased the reluctance to share data amongst South African researchers. A lack of funding and financial investment in physical infrastructure (i.e. power and the Internet) are contributing factors to the challenges in data availability and accessibility.^21^ Similarly, a Zimbabwean study discovered that persistent power challenges may be a factor in data sharing.^32^ These struggles are exacerbated by the current inequities in the global research community, which largely excludes researchers from LMICs from actively participating in the progression of science, where they are often relegated to the role of data generator, instead of published author.^33^ It is important that researchers and scientists are provided with the necessary resources and government support to reinforce their data-sharing processes.

Furthermore, Skelly and Chiware^34^ proposed that future policies define the roles of international research funders, journal publishers and inter-institutional and country collaborators to ensure equitable data custodianship in African-generated research. Data sharing is an important component of scientific investigation that should always strive to uphold the rights and interests of all stakeholders.^34^ This underscores the need for organisations and institutions to have data governance mechanisms in place, such as data management plans and policies that encapsulate ethical data-sharing practices.^35,36^

Whilst the focus of this paper is not on big data from commercial endeavours, one must note that data regulations govern both commercial and non-commercial big data. Although the difference between commercial and research big data lies in the motive for collecting and analysing data, where private information is involved, both commercial and research entities must treat data with care to ensure good governance.^37^ The Organisation for Economic Cooperation and Development (OECD) refers to data governance as the:

…diverse arrangements, including technical, policy, regulatory or institutional provisions, that affect data and their cycle (creation, collection, storage, use, protection, access, sharing and deletion) across policy domains and organisational and national borders.^38^

For the purposes of this paper, we define data governance as frameworks and policies that regulate data use, collection, storage or management, protection and sharing. Whilst some SSA countries have such frameworks in place, others still lag behind.^35,39–41^

One concern is that some countries may be transferring or sharing data without the existence of legislation, institutional policies or frameworks and good data management standards.^35^ Good data governance supports the generation of high-quality data and the preservation of control over data. South Africa’s *Protection of Personal Information Act* (POPIA)^42^ is an example of a firm privacy and security law as it closely resembles Europe’s General Data Protection Regulation (GDPR).^43^ In addition, Data Access Committees (DACs) have been shown to play an essential role in improving data governance within the context of research as they are able to approve or disapprove data access requests after deliberation and consideration of the potential benefit and harm to the individuals from whom the data were sourced, their communities, researchers and other stakeholders.^44^

Considering the big data revolution in the African region, continental researchers and scientists must reflect on data governance and regulation, and what it means to establish effective support systems for the management of large data sets.^34^ Whilst a growing body of global research has explored the practices and perceptions of researchers and scientists related to data governance and data protection policies and frameworks, there are limited studies on this phenomenon across SSA. Our study, therefore, aimed to address this gap by investigating the perceptions and experiences of researchers and scientists on data governance and data protection policies in SSA. In this paper, we present and discuss our major findings from data use and reuse, data practices, data management support, data sharing and data protection. Finally, we offer recommendations to strengthen data governance and facilitate responsible data sharing in SSA.

## Methods

### Study design and sampling

We conducted a descriptive cross-sectional online survey with both quantitative and qualitative components with 160 researchers and scientists representing 43 SSA countries from June 2022 to September 2022. The population was selected based on the profession of the participants as a researcher or scientist involved in data-intensive research in SSA. We recruited our sample through a purposive selection of the professional networks of Stellenbosch University’s Centre for Medical Ethics and Law across SSA and used a snowballing technique for further recruitment. We also identified potential participants through a desktop search based on their profession. The survey was directly emailed to those who fit the field of study, and they were invited to participate in their personal capacity. The European and Developing Countries Clinical Trials Partnership research network and Stellenbosch University’s Faculty of Medicine and Health Sciences’ Marketing and Communications newsletters were useful platforms to invite researchers and scientists to participate in the survey. Respondents were invited to anonymously participate in an online survey through Research Electronic Data Capture (REDCap). All respondents provided voluntary electronic consent.

### Survey instruments

The questionnaire was designed electronically using REDCap following a review of the current literature related to data sharing and data governance amongst scientists and researchers, and in consultation with experts in the field of big data research (see the Supplementary material). The face validity of the survey instrument was assessed by piloting the questionnaire with six data scientists and researchers. Minor amendments were made to produce the final version of the questionnaire before its circulation amongst respondents. These amendments included improving the language to enhance the ease of understanding and restructuring ambiguous questions. The questionnaire consisted of 16 closed-ended questions and three open-ended questions addressing demographic characteristics, respondents’ perspectives on data use and reuse, data management, data sharing and the use of others’ data. Regarding the open-ended qualitative aspect of the study, three questions were asked to briefly explore respondents’ thoughts on data protection steps, data use agreements and any additional comments they wished to add. The data collection tool was developed in English and further translated and localised into French and Portuguese by an academic institution’s language centre to cater for African Francophone and Lusophone countries. Data were collected through REDCap using mostly pre-defined categorical responses that did not require cleaning. The age category (not reported in our study) was missing in 91 (57%) of the respondents. This field was the only one that was not completed by all respondents. All 160 responses received were included in the analysis.

### Data analysis

Data were exported from REDCap to Statistical Package for Social Sciences (SPSS) (version 28) for analysis. Descriptive statistics were used to describe quantitative data using frequencies and/or percentages in tables and bar graphs. For the meaningful interpretation of the survey responses, questions presented on a five-point Likert scale as strongly disagree, disagree somewhat, neither agree nor disagree, agree somewhat and strongly agree were collapsed into three simpler categories: disagree, neither agree nor disagree, and agree.

In terms of the qualitative component of the study, a trained researcher manually analysed the responses to the open-ended questions using thematic analysis. The researcher first familiarised herself with the responses before identifying and creating codes. Thereafter, she identified patterns or recurring responses in the data. Quotations extracted from the data are included in the paper to illustrate findings from the participants’ perspectives. A manual method of analysis was employed due to the small volume of qualitative data that emerged from the three open-ended questions.^45^

### Ethical aspects

Research integrity was maintained throughout the study and participation in the research remained entirely voluntary. This survey was a minimal-risk study as the questionnaires involved a factual enquiry with educated and empowered respondents who had full capacity to consent or decline participation. The sample was approached in their individual capacities and respondents consented in their personal capacities. Ethics approval was granted by the Health Research Ethics Committee of the Faculty of Medicine and Health Sciences (reference no: N22/03/028) at Stellenbosch University, South Africa.

## Results

### Demographic information

**Figure 1:**
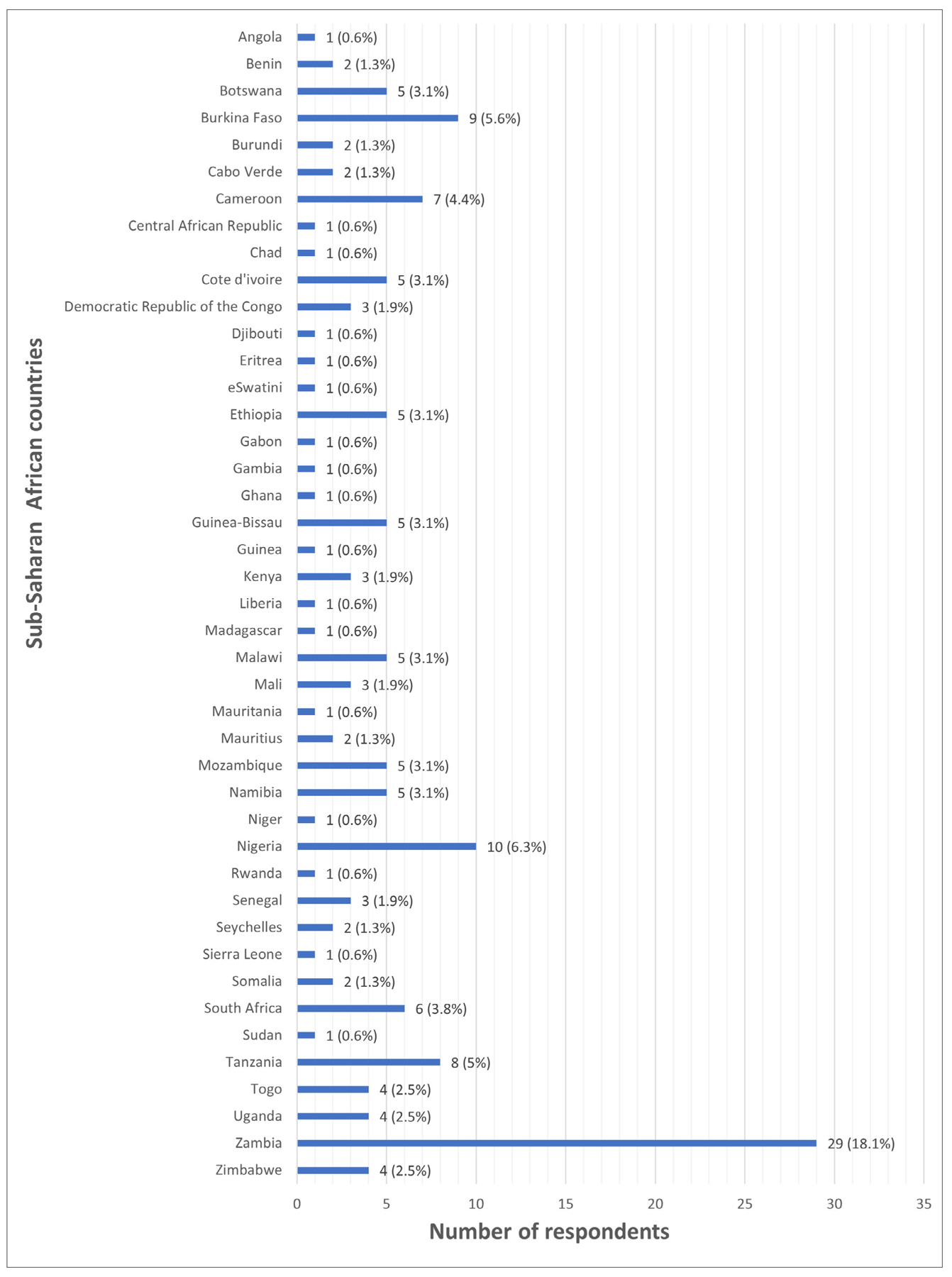
Number of respondents across the different sub-Saharan African countries.

In total, 160 individuals responded and completed the online survey. The respondents represented 43 of the 49 SSA countries, with 16 countries having at least one respondent (Figure 1).

Most respondents (68.8%) identified as male and were highly educated, with 60% having completed a doctorate, 52.5% being employed within academia and more than two-thirds (79.5%) self-identifying as researchers or scientists (Table 1).

### Data use and reuse

Most respondents reported generating their own data (76.3%) and described the sort of data that they worked with most often as research and academic data (58.8%), public health data (55%) or clinical health service data (37.5%) (Table 2).

**Figure 2:**
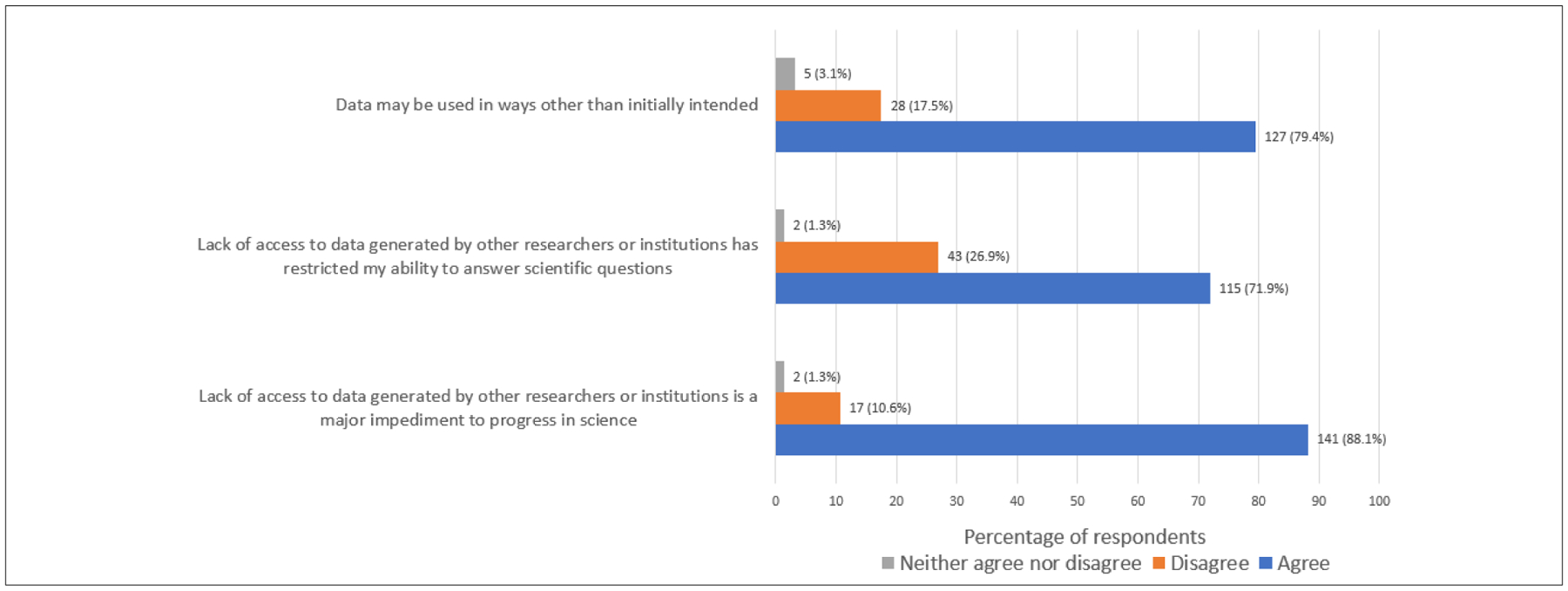
Perspectives on the reuse of data.

Regarding the reuse of data, a great number of respondents (88.1%) perceived the lack of access to data generated by other researchers and scientists or institutions as an impediment to scientific progress, and 71.9% reported facing limitations in answering scientific questions as a result thereof (Figure 2).

### Data practices

**Figure 3:**
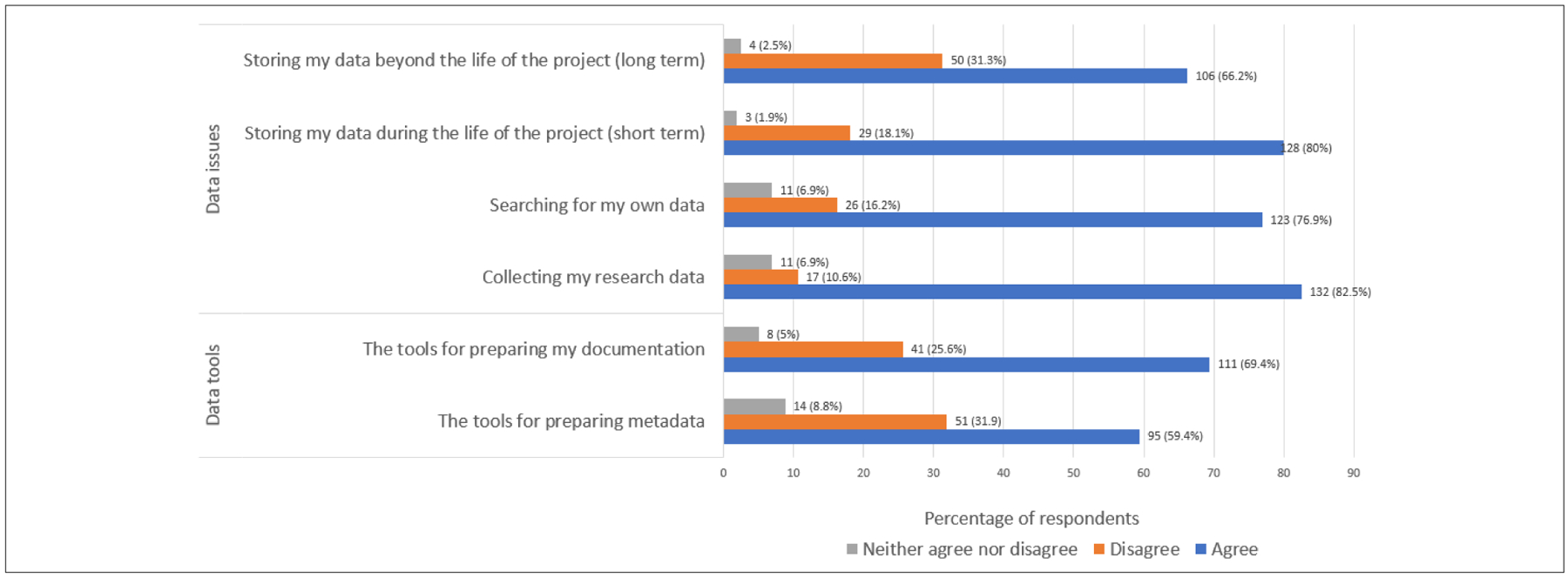
Satisfaction with data practices.

Data practices focused on the satisfaction rate of respondents’ processes used in collecting, searching for and storing their data. Most respondents reported satisfaction with their institutional processes for long- and short-term data storage (66.2% and 80%, respectively) (Figure 3). Data governance covers an important aspect of collecting and identifying data. Most respondents were satisfied with their current processes for the initial part of the research and data life cycle, which included searching for their data (76.9%) and collecting their data (82.5%). Respondents also reported satisfaction with the data tools used for the preparation of documentation (69.4%) and metadata (59.4%).

**Figure 4:**
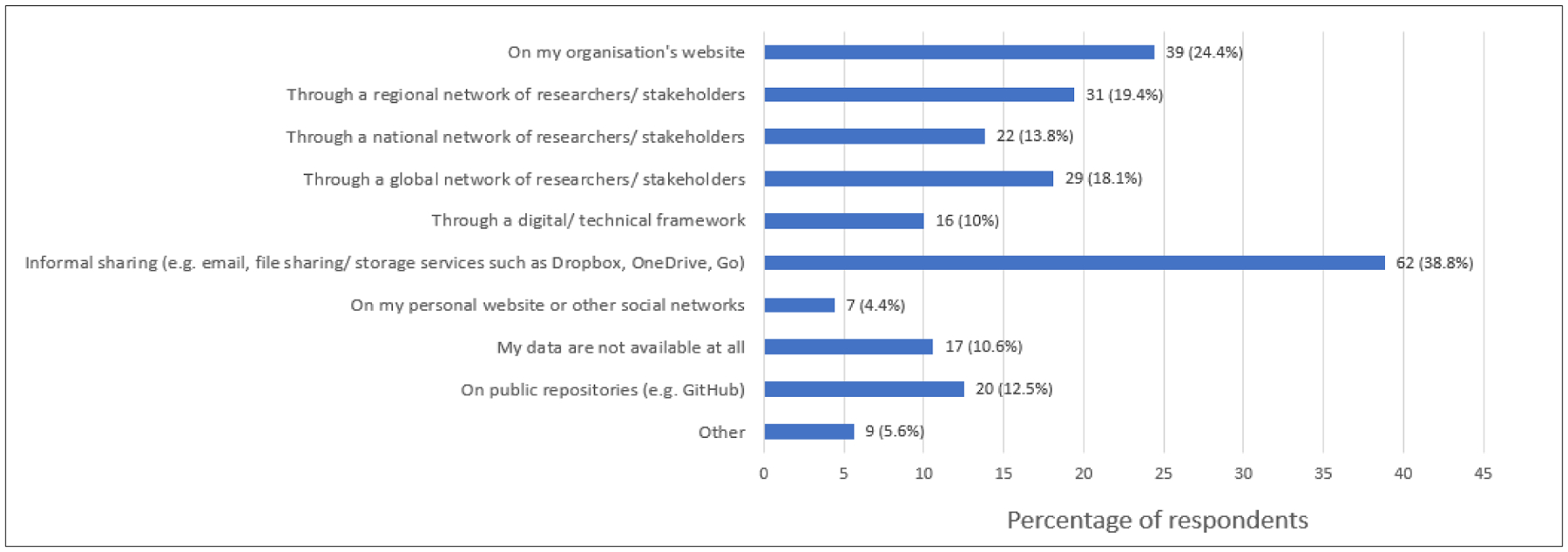
Data-sharing practices.

Just over a third (38.8%) of respondents indicated that most of their data were shared informally via emails and file-sharing or storage services such as Dropbox, OneDrive and Google Drive (Figure 4).

### Data management support

Our survey questions on data management support assessed the satisfaction rate of respondents concerning the level of support provided by their organisations in managing their data during and beyond the research project’s planning stage. Most respondents (75.7%) expressed satisfaction with the processes for managing their data, and 64.4% were satisfied with their institutional data management and/or governance plans (Table 3). The agreement rate for institutional or organisational support for data analysis during the life cycle of the project was higher over the short term (63.7%) than over the long term (53.1%).

Over half the respondents reported receiving the necessary tools and technical support for data management during (63.1%) and beyond (55%) the life cycle of the project. Just under half the respondents (40.6%) indicated receiving no training on practices for data management from their organisations or projects. Our results indicate that the provision of funds to support data management during the life cycle of a research project is higher (54.4%) than support beyond the life cycle of the research project (51.8%). These findings highlight the need for organisations or institutions to provide support or fund research data management and related infrastructure for researchers and scientists.

### Data sharing

**Figure 5:**
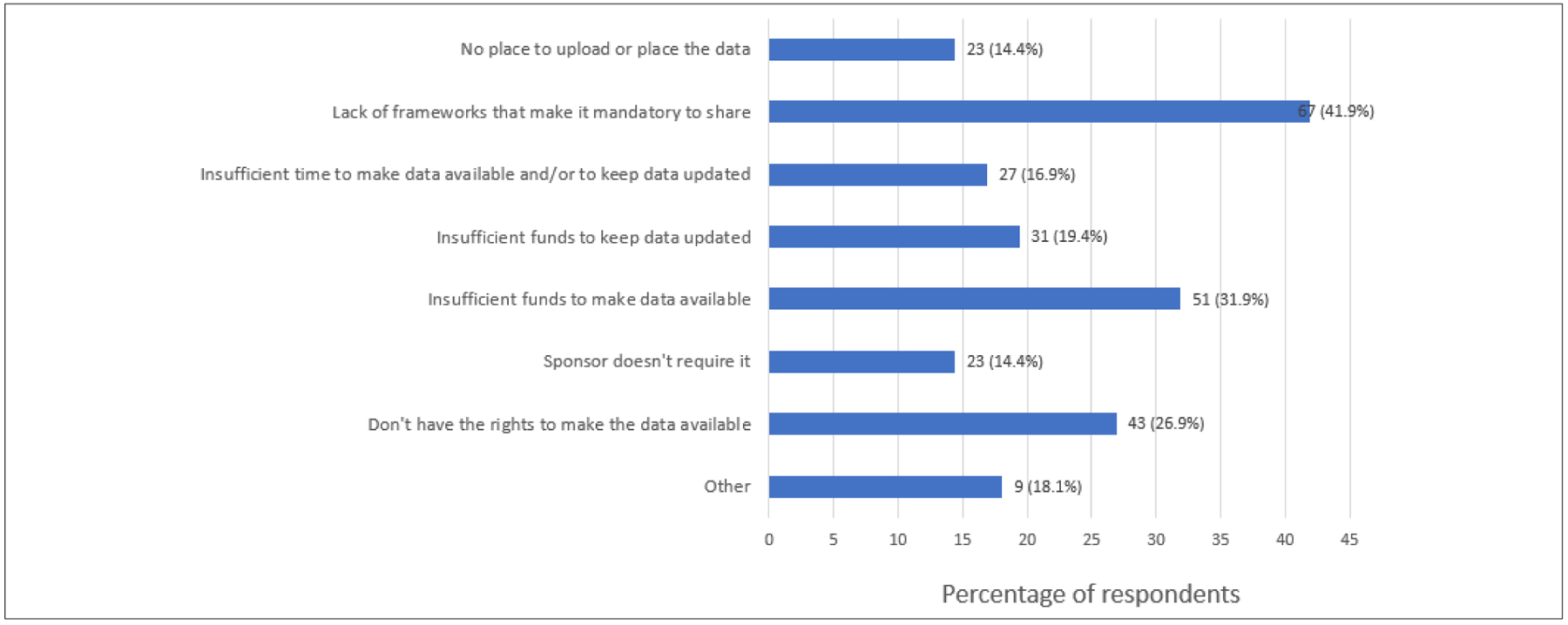
Reasons for not making data electronically available.

The lack of available frameworks for the mandatory sharing of data was found to be the most prominent reason (41.9%) for researchers and scientists across SSA countries to not make their data electronically available. This was followed by insufficient funds to make data available (31.9%) and not having the right to make the data available (26.9%) (Figure 5).

Almost all respondents (91.9%) agreed that they would use data sets of other researchers and scientists if these were easily accessible, and they would be willing to reciprocate (Table 4). Interestingly, most respondents (83.8%) reported a willingness to deposit some, but not all their data, into a public data repository lacking restrictions. This reported willingness to make data available increased when privacy and ethical conditions were applied (88.2%), as well as when there were conditions on governance and regulation on access (88.2%). This finding emphasises the importance of appropriate policies and governance mechanisms for data repositories to promote data sharing among scientists and researchers.^46^

Furthermore, most respondents were satisfied with exchanging their data for co-authorship on publications (89.4%) and the opportunity to collaborate on projects (77.6%).

Almost all respondents (94.4%) agreed on the importance of having their data cited by other researchers and scientists. Just over half the respondents (52.5%) were satisfied with exchanging their data for royalties, while others (41.3%) agreed to exchanging their data for commercialisation purposes (Table 5). Regarding their perspectives on using and sharing others’ data, the majority of respondents were satisfied (95.6%) with following ethical principles when using data from other researchers and scientists (Table 6). Most respondents were satisfied with offering co-authorship on publications in exchange for using other researchers’ and scientists’ data (77.5%) and the opportunity to collaborate on the project when using their data (93.1%). Over half the respondents (53.1%) disagreed with paying profits to other researchers and scientists to commercialise their data. Nearly two-thirds of the respondents (65.6%) were not keen on commercialising their data without profits (Table 7).

### Data protection

Through open-ended questions, respondents were asked about their data protection practices during the sharing of data. Most respondents reported not following any particular data protection steps, whilst others followed technologically based safety measures. Of those who indicated the use of protective measures, encryption, password-protected devices and Internet security (backups and firewalls) were included.

Electronic data: secure platforms/protocols are used, data is encrypted, tools may have multilayer verification steps and PINs. Preceded by training in human subjects’ protection, ethics in research.

[Country 2]

Confidentiality and anonymisation of information were other approaches supported by respondents.

The data should be protected confidentially to the benefit of both researchers and scientists and subjects as required in the scientific community.

[Country 5]

Data management, access and sharing policies were also identified as vital in data protection.

The one requesting the data has to write a formal email or complete the form in the institution drive stating why he/she needs the data and then sign a form. Thereafter, after noting the reason why he/she needs the data, partial rights to access data can be granted.

[Country 20]

DACs act as a gatekeeper for the data I generate. They review data access proposals and either grant or reject access based on the merit of the proposals. My data is accessed under the Fort Lauderdale rules of engagement, whereby there is a 2- to 3-year embargo for me to publish the data before public access is granted.

[Country 22]

Respondents reported using various data agreements when sharing data to protect data ownership rights and/or the privacy or sensitivity of the data. These included memoranda of understanding (MoUs), non-disclosure agreements, DTAs and MTAs. In addition, data licensing agreements and copyright clauses were reported as important sources of data protection used. Some respondents indicated the frequent use of traditional ethics guidelines provided by their respective research ethics committees when ensuring data protection during data sharing. Whilst consent processes are vital to data sharing, another layer of protection is needed to ensure that data are adequately protected, such as pseudonymisation and encryption.^47^

Consumers of data are required to sign non-disclosure agreements with confidentiality statements that they must adhere to when using protected data.

[Country 7]

Respondents referred to DACs, the GDPR^43^ and the Règlement Sanitaire International (RSI)^48^ (International Health Regulation, 2005) for guidance regarding data protection. On the other hand, some respondents revealed that they do not use any data protection agreements.

## Discussion

This study highlights the practices and perspectives of researchers and scientists in SSA countries regarding data sharing and data governance. Awareness of data protection policies and frameworks used in data governance was also explored. Respondents appeared relatively satisfied with their data storage processes, yet 40% indicated that their organisations or institutions did not have a formally established process for storing data beyond the life cycle of the project. There was less satisfaction with data management support; this challenge was experienced with respect to institutional support for data analysis, tools and technical issues. Again, long-term support appeared to be lacking. This finding is similar to that of Tenopir et al.,^6,7^ who reported that short-term storage solutions provide researchers and scientists with a degree of closeness to their data during the project life cycle. We also found that more than half of the respondents were satisfied with the available tools used for documentation preparation, whilst over a third of the respondents were dissatisfied with the tools used for preparing their metadata. This correlates with the findings of another study^7^ in which respondents were also dissatisfied with the tools used for preparing their metadata. This could suggest that there is a need for adequate tools to assist SSA researchers and scientists to facilitate and enhance their use and management of data.

Although most respondents were satisfied with the process of managing their data, 40.6% disagreed that their organisation provides training on best practices for data management. This could be related to a lack of resources, chronic under-investment in universities and institutions and suboptimal research training and mentorship in SSA.^49,50^ This unmet need for training in data management has been previously documented.^51,52^ Integrating data management into research methods coursework was suggested as a possible approach for encouraging best practices amongst researchers and scientists.^53^ With the growing adoption globally of big data, SSA researchers and scientists must be trained to harness big complex data sets to find solutions to scientific problems. Funding was another issue raised by respondents, with more than half indicating that their organisations did not provide the necessary funds to support data management beyond the life cycle of the project. These findings are similar to those of Tenopir et al.^6^ in which 59% of respondents indicated a lack of financial support for data management beyond the life cycle of the project. It will be crucial for organisations and institutions to invest and have sustainable funding for data management services in SSA. This has also been reported in other LMICs where the emphasis is on the importance of investment in data management.^54^

Open science and the sharing of data are essential for the advancement of science, and are seen as an important part of economic growth in Africa, which is burdened with dual public health and economic crises.^55,56^ Furthermore, from an ethical perspective, data sharing is a significant way to recognise the altruism and generosity of participants (for example, those from clinical trials) because it increases the utility of the data they provide and thus the value of their contribution.^57^ It was therefore important to explore the perspectives and practices of SSA researchers and scientists on data sharing. The majority of respondents reported that they had already shared their data. Lack of governance frameworks that make it mandatory to share data (41.9%) was one of the main reasons for not making data electronically available, followed by insufficient funds (31.9%). These reasons have also been reported as barriers to data sharing in LMICs, in African research institutions, as well as in institutions in Jordan.^54,58^ In the face of insufficient funding, Okafor et al.^59^ emphasised the importance of funding to institutionalise open science in Africa. The fact that open science for Africa is seen as a potential route to increased funding opportunities is particularly noteworthy. Researchers and scientists in Africa can gain visibility and funding from a broader group of potential funders by openly sharing their research findings.

Most respondents had positive views of data sharing, but 40.6% indicated a need to restrict all their data when placed in a public data repository.

This could be because there are either ethical issues or concerns about commercialisation. Most of the respondents also agreed to sharing their data, provided that the condition for sharing is to receive proper citation credit, co-authorship and an opportunity to collaborate. The respondents did not differ much in their perspectives on using others’ data. These findings support previous studies, where citation credit, co-authorship and an opportunity to collaborate were amongst the conditions and motivations for sharing and using others’ data.^6,34,60,61^ In contrast, some studies reported that African counterparts seem to be largely motivated by altruistic means for data sharing, such as emphasising the public benefit or the good of sharing knowledge and data.^34,62^ Nevertheless, the findings could suggest that African countries are gradually becoming familiar with the significance of data sharing and its impact on their researchers’ and scientists’ careers, which is different from several years ago.^63^ It would be useful for institutions or organisations to encourage data citation as a central data-sharing practice, and for researchers and scientists to be given co-authorship and collaboration in exchange for data sharing, taking authorship requirements into account.

It has been suggested that, in order to be eligible for co-authorship, a person must have made a significant contribution to the work (i.e. original acquisition, quality control and data curation) and be accountable for all aspects of the accuracy and integrity of the data provided, as well as ensure that the available data set adheres to the FAIR Guiding Principles.^12,64^ However, some studies have argued that co-authorship in exchange for data is a rather contentious issue, as it could be perceived as being potentially unethical.^65^ In addition, Hood and Sutherland^66^ further assert that author-type metrics, which are the gold standard for measuring scientific progression and success, are detrimental to scientific development. Hence, there is a need to develop different reward systems, whereby the output of data sets and data-index citations are collectively viewed as a measure of researcher growth and progression, instead of over-reliance on the number of publications or data-index citations. This shift in the reward system will greatly facilitate data sharing, especially in LMICs.^66^

Interestingly, respondents had different perspectives on the commercialisation of shared data, with half not agreeing to exchange others’ data for commercialisation purposes. These findings differ from those of a Malaysian study^67^ which found that 90% of the surveyed researchers and scientists were interested in commercialising their research. Our respondents’ views may have differed because some work with data (i.e. genetic information) that present significant dilemmas in the context of privacy and consent.^68^ Most respondents indicated that they do not use any data protection steps when sharing data other than using technologically based safety measures (e.g. password protection or encryption methods). This is concerning as it suggests that researchers and scientists are still making use of suboptimal or mediocre data practices, placing their data at risk for misuse or theft, amongst other concerns.^7^ There is a need to encourage researchers and scientists in the African context to prioritise good data practices by storing and sharing data in repositories.^7^ This can be accomplished by changing researchers’ negative perceptions around repositories by educating them on the standards and criteria of data repositories (increased security), as well as the benefits, such as adequately prepared metadata and the discoverability of the data.^57^ Europe has adopted a common legal, governance, data quality and operability framework to facilitate access to and reuse of health data.^69^

Another aspect of our findings was that respondents mentioned various data agreements they used when sharing data. These included DTAs, MTAs and MoUs. However, some of the respondents mentioned that they lacked such agreements. A common suggestion to improve these challenges included the development of DACs. Such committees balance issues of data ownership and foster data governance through their ability to approve or disapprove data access requests.^44,69^ This poses a question as to how SSA researchers and scientists share their data without the existence of policies or frameworks in their institutions or organisations. It is important to note that the lack of governance frameworks was the top reason respondents did not share their data. This has also been reported in the literature, where the lack of policy and guideline frameworks at institutional and national levels is one of the reasons for African researchers and scientists not sharing their data.^34^ About 18 SSA countries (including South Africa and Kenya) have a comprehensive data protection law that is currently in effect.^70^ Considering the current advancements in digital technologies, SSA countries must implement data protection policies and frameworks that are a contextual fit, as this could provide assurances and confidence amongst researchers and scientists that measures are in place to secure their data sets during the sharing or transfer of data.

Furthermore, having policies or frameworks in place could encourage researchers and scientists in SSA to make their data electronically available. Despite the benefits of data sharing promoted by funders and journals, the volume of shared data remains low.^71^ Buy-in from and support for institutions or organisations to establish data-sharing policies that specify aims and data request procedures may be required. Cheah^71^ advised that the aims should be aligned with the institutional or organisational aims, as this would help researchers and scientists maximise the use of their data for primary and secondary analyses. In addition, having a data-sharing policy could put an institution or organisation in a better position when applying for funding and submitting manuscripts for publication.^71^ Nevertheless, there is a need for engagement or collaboration between researchers and scientists, their funders and institutions or organisations to find creative solutions that could enhance responsible and sustainable data governance.

Overall, the survey found that researchers and scientists were optimistic about data sharing, storage, data management support and reuse. Many researchers and scientists across SSA are using various types of data agreements and security measures during data sharing, whilst other researchers lack such tools, approaches and data protection policies and frameworks that promote safe data sharing. The study findings have been interpreted and discussed in light of the current available literature. When compared to the findings of previous global studies^6,7,34,54,58,60,61^, our findings were similar and comparable in terms of data practices, data management support and data-sharing practices. However, some differences emerged in the perspectives of data sharing for commercialisation purposes.^67^

### Study limitations

The study is not without its limitations, which should be considered when interpreting the findings. There was a predominance of respondents from Zambia, Nigeria, Burkina Faso, Tanzania, Cameroon and South Africa in comparison with other SSA countries. This could be because email access was better in these countries. A consistent and salient finding across the comparison of responses from these six SSA countries with the highest number of responses was that most views were aligned – apart from some recurrent variations regarding organisational involvement in data activities and conditions of fair exchange. Based on previous experience with conducting research in SSA, obtaining a response to surveys is challenging, so we aimed to get a minimum of one response per country. Access to the Internet and email is inequitable in various settings in Africa.^72^ It is with significant effort that we were able to elicit responses from 43/49 SSA countries. The sample size was relatively small and may not represent all researchers and scientists in SSA countries. Future studies could include a larger sample across SSA countries so that the findings could be generalisable to the overall research population. However, data collection would take significantly longer than 4 months, given the challenges with responsiveness and Internet or email access that exist on the continent. Those respondents that did not complete the survey might have felt that the survey was too long. Despite these limitations, this study has provided a broad overview of important practices and perspectives on data governance amongst a sample of researchers and scientists in SSA, and has informed the qualitative phase of our study, in which we conducted in-depth interviews.

## Conclusion

Data sharing is generally recognised as a public good that increases the diversity of research data. Most respondents demonstrated a positive attitude towards data sharing and were willing to share at least some of their data, conditional upon robust governance with certain restrictions. In addition to funding, there is a need for the institutional support of data management, robust data protection legislation and appropriate policies to guide and promote data sharing in SSA countries. Given that DTAs vary between projects and countries, having standardised templates for DTAs and data use agreements would expedite sharing agreements between research collaborators. This will enable researchers and scientists, their funders, journals and institutions to collaborate and promote sustainable data sharing on the continent. In this context, sustainable data sharing includes providing ethical incentive structures for researchers and scientists who are willing to share their data, as well as tangible infrastructure to facilitate such sharing. Capacity development in data governance for researchers and scientists is sorely needed – and relevant knowledge transfer between SSA countries should be facilitated. Perceived and actual risks of commercialisation require further exploration.

## Supplementary Material

Survey Template

## Figures and Tables

**Table 1:** Characteristics of survey respondents (*N* = 160)

Characteristic	*n* (%)
**Job title**	
Business analyst	6 (3.8)
Data scientist	13 (8.1)
Developer	1 (0.6)
Researcher	116 (72.5)
Other	24 (15.0)
**Gender**	
Male	110 (68.8)
Female	50 (31.3)
**Education/qualification**	
Bachelor’s degree	7 (4.4)
Honours degree	3 (1.9)
Master’s degree	50 (31.3)
Doctoral degree	96 (60)
Other	4 (2.5)
**Employment by sector**	
Academia	84 (52.5)
Government or public sector	33 (20.6)
Commercial	2 (1.3)
Not-for-profit organisation	37 (23.1)
Other	4 (2.5)

**Table 2:** Data use among respondents (*N* = 160)

Which term/s best describe/s the type of data you use? (Multiple selections applicable)	*n* (%)
Public health data	88 (55)
Clinical health services data	60 (37.5)
Research and academic data	94 (58.8)
Environmental data	19 (11.9)
Behavioural and socio-economic data	24 (15)
Health capabilities data	23 (14.4)
Information and communication technologies industry data	15 (9.4)
Individual and group data	20 (12.5)
Non-health data	9 (5.6)
Experimental	23 (14.4)
Interviews	28 (17.5)
Observational	29 (18.1)
Other	3 (1.9)
**Do you own or generate the data you work with?**	
Yes	122 (76.3)
No	38 (23.8)

**Table 3:** Organisational involvement in data issues (*N* = 160)

	Agree *n* (%)	Disagree *n* (%)	Neither agree nor disagree *n* (%)
I am satisfied with the process of managing my data	121 (75.7)	30 (18.7)	9 (5.6)
I am satisfied with my institution’s data management and/or governance plan	103 (64.4)	47 (29.4)	10 (3.3)
My organisation or project has a formal established process for supporting data analysis during the life of the project (short term)	102 (63.7)	50 (31.3)	8 (5)
My organisation or project has a formal established process for supporting data analysis beyond the life of the project (long term)	85 (53.1)	66 (41.3)	9 (5.6)
My organisation or project has a formal established process for storing data beyond the life of the project (long term)	87 (54.4)	65 (40.7)	8 (5)
My organisation or project has a formal established process for managing data during the life of the project (short term)	99 (61.9)	52 (32.6)	9 (5.6)
My organisation or project provides the necessary tools and technical support for data management during the life of the project (short term)	101 (63.1)	52 (32.6)	7 (4.4)
My organisation or project provides the necessary tools and technical support for data management beyond the life of the project (long term)	88 (55)	64 (40)	8 (5)
My organisation or project provides training on best practices for data management	86 (53.8)	65 (40.6)	9 (5.6)
My organisation or project provides the necessary funds to support data management during the life of a research project (short term)	87 (54.4)	65 (40.6)	8 (5)
My organisation or project provides the necessary funds to support data management beyond the life of the project (long term)	71 (44.4)	83 (51.8)	6 (3.8)

**Table 4:** Conditions for data sharing (*N* = 160)

	Agree *n* (%)	Disagree *n* (%)	Neither agree nor disagree *n* (%)
I would use other researchers’ data sets if their data sets were easily accessible	147 (91.9)	9 (5.7)	4 (2.5)
I would equally reciprocate data sharing when data are shared with me	147 (91.9)	10 (6.2)	3 (1.9)
I would be willing to place at least some of my data into a public data repository with no restrictions	134 (83.8)	20 (12.6)	6 (3.8)
I would be willing to place all my data into a public data repository with no restrictions	88 (55)	65 (40.6)	7 (4.4)
I would be willing to make my data available if I could place privacy and ethical conditions on access	141 (88.2)	12 (7.5)	7 (4.4)
I would be more likely to make my data available if I could place conditions of governance and regulation on access	141 (88.2)	13 (8.1)	6 (3.8)
I would be willing to share data across a broad group of researchers who use data in different ways	139 (86.9)	18 (11.2)	3 (1.9)
It is important that my data are cited when used by other researchers	151 (94.4)	3 (1.9)	6 (3.8)
I am satisfied with exchanging my data knowing that secondary data will be retrieved and shared from my original data set, and then allowing those data to be shared	136 (85)	15 (9.4)	9 (5.6)
I am satisfied with exchanging my data if I know they will be used ethically	148 (92.5)	6 (3.7)	6 (3.8)
I am satisfied with exchanging my data for co-authorship on publications	139 (86.9)	16 (10.1)	5 (3.1)
I am satisfied with exchanging my data for formal acknowledgement in all disseminated work using those data	133 (83.1)	22 (13.8)	5 (3.1)
I am satisfied with exchanging my data for formal citation in all disseminated work using those data	143 (89.4)	13 (8.1)	4 (2.5)
I am satisfied with exchanging my data for the opportunity to collaborate on the project	140 (77.6)	14 (8.8)	6 (3.8)

**Table 5:** Conditions for data sharing related to commercialisation (*N* = 160)

	Agree *n* (%)	Disagree *n* (%)	Neither agree nor disagree *n* (%)
I am satisfied with exchanging my data for royalties	84 (52.5)	69 (43.1)	7 (4.4)
I am satisfied with exchanging my data for commercialisation purposes with profits	66 (41.3)	86 (53.8)	8 (5)
I am satisfied with exchanging my data for commercialisation purposes without profits	72 (45)	80 (50.1)	8 (5)
I am satisfied with exchanging my data for the recovery of a portion of the costs of data acquisition, retrieval or provision	88 (55.1)	62 (38.7)	10 (6.3)

**Table 6:** Using others’ data (*N* = 160)

	Agree *n* (%)	Disagree *n* (%)	Neither agree nor disagree *n* (%)
I am satisfied with extracting secondary data from the primary data of other researchers and then share those data	132 (82.6)	25 (15.6)	3 (1.9)
I am satisfied with following ethical principles when using other researchers’ data	153 (95.6)	5 (3.1)	2 (1.3)
I am satisfied with offering co-authorship on publications in exchange for using other researchers’ data	124 (77.5)	32 (20.1)	4 (2.5)
I am satisfied with formally acknowledging other researchers in all disseminated work using their data	148 (92.6)	8 (5.1)	4 (2.5)
I am satisfied with formally citing other researchers in all disseminated work using their data	150 (93.8)	6 (3.8)	4 (2.5)
I am satisfied with offering other researchers the opportunity to collaborate on the project when using their data	149 (93.1)	8 (5)	3 (1.9)

**Table 7:** Using others’ data related to commercialisation (*N* = 160)

	Agree *n* (%)	Disagree *n* (%)	Neither agree nor disagree *n* (%)
I am satisfied with paying royalties to use other researchers’ data	75 (46.9)	80 (50)	5 (3.1)
I am satisfied with paying profits to other researchers to commercialise their data	66 (41.3)	85 (53.1)	9 (5.6)
I am satisfied with commercialising other researchers’ data without paying them profits	48 (30)	105 (65.6)	7 (4.4)
I am satisfied with compensating a portion of the costs of data acquisition, retrieval or provision to other researchers when using their data	94 (58.8)	57 (35.6)	9 (5.6)
