# Supplementary material for: Data sharing and data governance in sub-Saharan Africa: Perspectives from researchers and scientists engaged in data-intensive research
Source: S Afr J Sci. Author manuscript; Available in PMC 2024 Oct 31. (PMC11526389; doi:10.17159/sajs.2023/15129)
Supplement: Survey Template [file NIHMS1997167-supplement-Survey_Template.pdf]

SUPPLEMENTARY MATERIAL TO: Kabanda et al. S Afr J Sci. 2023;119(5/6), Art. #15129

HOW TO CITE:
Kabanda SM, Cengiz N, Rajaratnam K, Watson BW, Brown Q, Esterhuizen TM, et al. Data sharing and data governance in sub-Saharan Africa: Perspectives from researchers and scientists engaged in data-intensive research [supplementary material]. S Afr J Sci. 2023;119(5/6), Art. #15129. https://doi.org/10.17159/sajs.2023/15129/suppl

---

Scientist/Researcher Survey

1 Please select the country that you currently work in or have worked in for most of your employment years.

- ◯ Angola
- ◯ Benin
- ◯ Botswana
- ◯ Burkina Faso
- ◯ Burundi
- ◯ Cabo Verde
- ◯ Cameroon
- ◯ Central African Republic
- ◯ Chad
- ◯ Comoros
- ◯ Côte d'Ivoire
- ◯ Democratic Republic of the Congo
- ◯ Djibouti
- ◯ Equatorial Guinea
- ◯ Eritrea
- ◯ eSwatini
- ◯ Ethiopia
- ◯ Gabon
- ◯ Gambia
- ◯ Ghana
- ◯ Guinea
- ◯ Guinea-Bissau
- ◯ Kenya
- ◯ Lesotho
- ◯ Liberia
- ◯ Madagascar
- ◯ Malawi
- ◯ Mali
- ◯ Mauritania
- ◯ Mauritius
- ◯ Mozambique
- ◯ Namibia
- ◯ Niger
- ◯ Nigeria
- ◯ Republic of the Congo
- ◯ Rwanda
- ◯ Sao Tome and Principe
- ◯ Senegal
- ◯ Seychelles
- ◯ Sierra Leone
- ◯ Somalia
- ◯ South Africa
- ◯ South Sudan
- ◯ Sudan
- ◯ Tanzania
- ◯ Togo
- ◯ Uganda
- ◯ Zambia
- ◯ Zimbabwe

	Please indicate your job title.	◯ Business Analyst (Project Manager; Product Manager; Program Manager)<br>◯ Data Scientist (Data Engineer; Data Analyst; Data Consultant)<br>◯ Developer (Software Engineer; Java Developer; Hadoop Developer; Software Developer)<br>◯ Engineer (Data Architect; DevOps Engineer; Solution Architect; Systems Engineer)<br>◯ Researcher<br>◯ Other
	If you selected other, please specify.	__________________________________
	Please indicate your age category.	◯ 18-29<br>◯ 30-39<br>◯ 40-49<br>◯ >50
2	Please specify your gender.	◯ Female<br>◯ Male<br>◯ Other
	What is the highest level of post-school education you have completed?	◯ Trade/ technical/ vocational training<br>◯ Bachelors degree<br>◯ Honours degree<br>◯ Masters degree<br>◯ Doctorate degree<br>◯ Other
4	If you selected other, please specify.	__________________________________
	Which one of the following best describes your primary work sector?	◯ Academic<br>◯ Government/Public sector<br>◯ Commercial<br>◯ Not-for-profit organisation<br>◯ Other
	If you selected other, please specify.	__________________________________

Which term/s best describe the type of data you use? (Check all that apply)	☐ Public health data (e.g. disease surveillance, immunization records, public health reporting, vital statistics, registries)<br>☐ Health services data (e.g. clinical records, EHR/EMR, prescribing, diagnostics, laboratory, insurance, healthcare providers & institutions, public health agencies, professional associations, pharma, biotech, health technology)<br>☐ Research and academia data (e.g. research institutes & networks, universities registries, genomics, omics, clinical trials, biobanks)<br>☐ Environmental data (e.g. climate, meteorological, transport, pollution, energy, geospatial)<br>☐ Behavioural and socioeconomic data (e.g. wellness, fitness, internet, social media, self monitoring, wearables, sensors, apps, mobile, loyalty cards, store transactions, location tracking, financial, education)<br>☐ Health capabilities data (policy, analytical and technological e.g. principles, aims, tools, governance, ethics, visualization, integration, prediction, modelling, synthesis, insight, storage, curation, extraction, interoperability, protection)<br>☐ Information and communication technologies (ICT) industry data (e.g. standards organizations, ICT businesses, telecommunication, security, analytics, marketing)<br>☐ individual and group data (e.g. citizens, consumers, patients, civil society, health agencies, regulators, ICT agencies, international organizations)<br>☐ Non-health data (e.g. migration data)<br>☐ Experimental (involving some degree of manipulation)<br>☐ Interviews<br>☐ Observational (no manipulation involved)<br>☐ Other
If you selected other, please specify.	__________________________________
Do you own or generate the data that you work with?	◯ Yes<br>◯ No
If some or all of your data are available to others, on which platforms are these data available and how much of it is made available? (Check all that apply)	☐ On my organization's website<br>☐ Through a regional network of researchers/ stakeholders<br>☐ Through a national network of researchers/ stakeholders<br>☐ Through a global network of researchers/ stakeholders<br>☐ Through a digital/ technical framework<br>☐ Informal sharing (e.g. email, file sharing/ storage services such as Dropbox, OneDrive, Google Drive, EMC, Azure and AWS)<br>☐ On my personal website or other social networks<br>☐ My data is not available at all<br>☐ On public repositories (e.g. GitHub)<br>☐ Other

If you selected other, please specify.

____

What kind of data protection steps have been taken into account when sharing the data?

____

What type of data use agreement is used when sharing the data to protect data ownership rights, and/or privacy/sensitivity of the data?

____

If your data are not available electronically to others, why not? (Check all that apply)

- ☐ Insufficient funds to make data available
- ☐ Insufficient funds to keep data updated
- ☐ Insufficient time to make data available and/or to keep it updated
- ☐ Lack of frameworks that make it mandatory to share
- ☐ No place to upload or place the data
- ☐ Sponsor doesn't require it
- ☐ Don't have the rights to make the data available
- ☐ Other

____

The following group of statements relates to how you collect and use research data. Tell us how much you agree with each statement:

	Disagree strongly	Disagree somewhat	Agree somewhat	Agree strongly	Not applicable
I am satisfied with the process for managing my data	○	○	○	○	○
I am satisfied with my institution's data management and/or governance plan	○	○	○	○	○
I am satisfied with the process for collecting my research data.	○	○	○	○	○
I am satisfied with the process for searching for my own data.	○	○	○	○	○
I am satisfied with the process for storing my data during the life of the project (short-term).	○	○	○	○	○
I am satisfied with the process for storing my data beyond the life of the project (long-term).	○	○	○	○	○
I am satisfied with the process for analysing my data (by myself or through a biostatistician).	○	○	○	○	○
I share my data with others.	○	○	○	○	○

Others can access my data easily.	◯	◯	◯	◯	◯
I am satisfied with the tools for preparing metadata.	◯	◯	◯	◯	◯
I am satisfied with the tools for preparing my documentation.	◯	◯	◯	◯	◯

The following group of statements relates to how your organisation is involved with your data. Tell us how much you agree with each statement:

	Disagree strongly	Disagree somewhat	Agree somewhat	Agree strongly	Not applicable
My organization or project has a formal established process for supporting data analysis during the life of the project (short-term).	◯	◯	◯	◯	◯
My organization or project has a formal established process for supporting data analysis beyond the life of the project (long-term).	◯	◯	◯	◯	◯
My organization or project has a formal established process for managing data during the life of the project (short-term).	◯	◯	◯	◯	◯
My organization or project has a formal established process for storing data beyond the life of the project (long-term).	◯	◯	◯	◯	◯
My organization or project provides the necessary tools and technical support for data management during the life of the project (short-term).	◯	◯	◯	◯	◯
My organization or project provides the necessary tools and technical support for data management beyond the life of the project (long-term).	◯	◯	◯	◯	◯
My organization or project provides training on best practices for data management.	◯	◯	◯	◯	◯
My organization or project provides the necessary funds to support data management during the life of a research project (short-term).	◯	◯	◯	◯	◯

My organization or project provides the necessary funds to support data management beyond the life of the project (long-term).	○	○	○	○	○

The following group of statements relates to your views on the use of data across your research field. Tell us how much you agree with each statement.

	Disagree strongly	Disagree somewhat	Agree somewhat	Agree strongly	Not applicable
Lack of access to data generated by other researchers or institutions is a major impediment to progress in science.	○	○	○	○	○
Lack of access to data generated by other researchers or institutions has restricted my ability to answer scientific questions.	○	○	○	○	○
Data may be used in other ways than initially intended.	○	○	○	○	○

The following group of statements relates to data sharing. Tell us how much you agree with each statement.

	Disagree strongly	Disagree somewhat	Agree somewhat	Agree strongly	Not applicable
I would be willing to place my data under embargo (restriction placed on data for limited period).	○	○	○	○	○
I am satisfied with other researchers placing their data under embargo.	○	○	○	○	○
I would be willing to equally reciprocate data sharing when data is shared with me.	○	○	○	○	○
I would use other researchers' datasets if their datasets were easily accessible.	○	○	○	○	○
I would be willing to place at least some of my data into a public data repository with no restrictions.	○	○	○	○	○
I would be willing to place all of my data into a public data repository with no restrictions.	○	○	○	○	○

I would be more likely to make my data available if I could place privacy and ethical conditions on access .	○	○	○	○	○
I would be more likely to make my data available if I could place conditions of governance and regulation on access	○	○	○	○	○
I am satisfied with my ability to integrate data from disparate sources to address research questions.	○	○	○	○	○
I would be willing to share data across a broad group of researchers who use data in different ways.	○	○	○	○	○
It is important that my data are cited when used by other researchers.	○	○	○	○	○
It is appropriate to combine shared datasets from different researchers into one dataset and then publish that dataset.	○	○	○	○	○

The following group of statements relates to conditions of fair exchange for the use of your data. Tell us how much you agree with each statement.

	Disagree strongly	Disagree somewhat	Agree somewhat	Agree strongly	Not applicable
I am satisfied with exchanging my data knowing that secondary data will be retrieved from my original dataset and then allowing that data to be shared.	○	○	○	○	○
I am satisfied with exchanging my data if I know it will be used ethically.	○	○	○	○	○
I am satisfied with exchanging my data for royalties.	○	○	○	○	○
I am satisfied with exchanging my data for commercialisation purposes with profits.	○	○	○	○	○
I am satisfied with exchanging my data for commercialisation purposes without profits.	○	○	○	○	○

I am satisfied with exchanging my data for co-authorship on publications.	○	○	○	○	○
I am satisfied with exchanging my data for formal acknowledgement in all disseminated work using that data.	○	○	○	○	○
I am satisfied with exchanging my data for formal citation in all disseminated work using that data	○	○	○	○	○
I am satisfied with exchanging my data for the opportunity to collaborate on the project.	○	○	○	○	○
I am satisfied with exchanging my data for the authority to approve/disapprove data disseminated in any format.	○	○	○	○	○
I am satisfied with exchanging my data for the recovery of a portion of the costs of data acquisition, retrieval or provision.	○	○	○	○	○
I am satisfied with exchanging my data for the opportunity to review the results and make suggestions or comments on data that could not be disseminated.	○	○	○	○	○
I am satisfied with exchanging my data for the right to approve/disapprove reprints of articles and/or digital resharing of articles that make use of my data.	○	○	○	○	○
I am satisfied with exchanging my data for a complete updated list of all products that make use of my data, including articles, presentations, educational materials, etc.	○	○	○	○	○

The following group of statements relates to conditions of fair exchange for the use of other researchers' data. Tell us how much you agree with each statement.

	Disagree strongly	Disagree somewhat	Agree somewhat	Agree strongly	Not applicable
I am satisfied with extracting secondary data from the primary data of other researchers' and then sharing that data.	○	○	○	○	○
I am satisfied with following ethical principles when using other researchers' data.	○	○	○	○	○
I am satisfied with paying royalties to use other researchers' data.	○	○	○	○	○
I am satisfied with paying profits to other researchers' to commercialise their data.	○	○	○	○	○
I am satisfied with commercializing other researchers' data without paying them profits.	○	○	○	○	○
I am satisfied with offering co-authorship on publications in exchange of using other researchers' data.	○	○	○	○	○
I am satisfied with formally acknowledging other researchers' in all disseminated work using their data.	○	○	○	○	○
I am satisfied with formally citing other researchers' in all disseminated work using their data.	○	○	○	○	○
I am satisfied with offering other researchers' the opportunity to collaborate on the project when using their data.	○	○	○	○	○
I am satisfied with offering other researchers' the authority to approve/disapprove data disseminated in any format when using their data.	○	○	○	○	○
I am satisfied with compensating a portion of the costs of data acquisition, retrieval or provision to other researchers' when using their data.	○	○	○	○	○

I am satisfied with offering other researchers' the opportunity to review the results and make suggestions or comments on any of their data that could not be disseminated.	○	○	○	○	○
I am satisfied with offering other researchers' the right to approve/disapprove article reprints and/or digital resharing of articles that use their data.	○	○	○	○	○
I am satisfied with offering other researchers' a complete updated list of all products that make use of their data, including articles, presentations, educational materials, etc.	○	○	○	○	○

Please share any additional comments, concerns, questions, or suggestions about your use of data?

____
